# Classification challenges of the 2019 revised International Standards for Neurological Classification of Spinal Cord Injury (ISNCSCI)

**DOI:** 10.1038/s41393-021-00648-y

**Published:** 2021-06-04

**Authors:** Steven Kirshblum, Mary Schmidt Read, Rüdiger Rupp

**Affiliations:** 1 Kessler Institute for Rehabilitation, West Orange, NJ USA; 2 Rutgers New Jersey Medical School, Newark, NJ USA; 3 Magee Rehabilitation/Jefferson Health, Philadelphia, PA USA; 4 Spinal Cord Injury Center, Heidelberg University Hospital, Heidelberg, Germany

**Keywords:** Neurology, Outcomes research

## Abstract

**Study design:**

Retrospective review of ISNCSCI datasets.

**Objectives:**

To discuss the correct classification of ISNCSCI datasets considered as challenging.

**Setting:**

International expert collaboration.

**Methods:**

The International Standards Committee of the American Spinal Injury Association (ASIA) receives challenging case scenarios regarding the International Standards for the Neurological Classification of Spinal Cord Injury (ISNCSCI). Among those cases received, sample cases representing different categories of typical classification difficulties were identified by members of the International Standards committee.

**Results:**

From the cases received, five sample cases were identified as representative for publication. These cases are related to the correct classification in the presence of non-SCI related conditions, the determination of motor zones of partial preservation in regions with no myotomes to test, the classification of the ASIA Impairment Scale in patients with substantial motor function below the motor level but no sacral sparing, the inclusion of non-key muscle functions in the classification of sensory incomplete individuals, and the correct classification of individuals with an amputation.

**Conclusion:**

Presenting cases with challenging classifications, along with responses and explanations, will serve spinal cord injury professionals to better understand and utilize the ISNCSCI classification. As the ISNCSCI endorsed by ASIA and the International Spinal Cord Society (ISCoS) evolves over time, such resources are important to clarify inquiries from the spinal cord injury community and to understand the rationale for revisions.

## Introduction

Since the introduction of the original Standards for the Classification of Spinal Cord Injuries by the American Spinal Injury Association (ASIA) in 1982 [1], the International Standards for the Neurological Classification of Spinal Cord Injury (ISNCSCI) has undergone a number of revisions [2–5], including most recently in 2019 as fully described in other articles in this issue. The ISNCSCI endorsed by ASIA and the International Spinal Cord Society (ISCoS) is the most widely used classification in the field of spinal cord injury (SCI) medicine, and describes the examination and definitions to be used for clinical and research purposes around the world.

The ASIA International Standards Committee often receives inquiries regarding the ISNCSCI. In January of 2019, a call for challenging cases to be submitted to the committee was communicated via e-mail to all ASIA membership. These questions are usually handled by the Chair of the committee along with other committee members to assure a consensus response, and then sent directly to the person(s) who posed the question. Previous cases, as well as other challenging questions regarding the ISNCSCI, have been published to serve as a resource for other SCI professionals to consult [6, 7].

In this paper, we describe five case scenarios that represent challenging questions with their explanations; the explanations include some of the Standard revisions introduced in 2019. The questions include (1) how to score a complicated case when a tagged score would make the difference between an ASIA Impairment Scale (AIS) C and D; (2) how to score the motor zone of partial preservation (ZPP) in the thoracic area with varied sensory scores; (3) can the sacral sparing definition be used to classify the injury as complete even if the individual can ambulate; (4) how to classify a case with non-key muscles documented; and (5) how to classify a case with an amputation above the suspected level of injury.

## Methods

Fifteen cases were submitted to the committee as a response to the call. These cases were related to problems with the classification of two spinal injuries, concomitant non-SCI conditions, amputations, not determinable AIS/Neurological Level of Injury (NLI) due to non-testable (NT) being documented in decisive segments, autonomous reactions on deep anal pressure (DAP), ambulatory AIS A patients, and other general challenging cases. Among those cases received by the International Standards Committee and responded to, sample cases representing different categories of typical classification difficulties were identified and included here. For each of the cases received, some committee members (including the authors) reviewed the questions. One member would draft a response that was evaluated by other review members. Any differences of opinion would be discussed until consensus was obtained. In all cases, there was unanimous agreement of the response.

## Results

### Challenging case 1

**Fig. 1:**
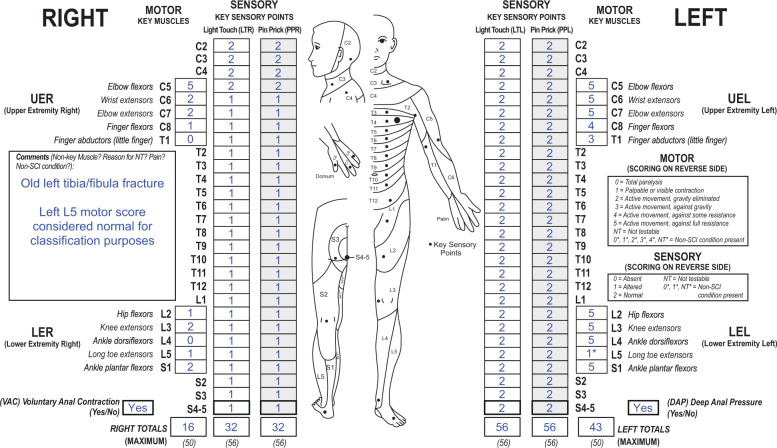
Case 1 with a non-SCI condition superimposed to the SCI. This individual has an unimpaired sensory function on the left side and a motor impairment of the left long toe extensor due to an old tibia fracture documented as 1* in the left L5 segment. Based on the clinical assumption, this asterisk (*)-tagged score should be considered as normal during classification, which is noted in the comments box.

With adoption of the 2019 revisions to the International Standards, what is the best way to classify a case when there is a myotome not grading as normal due to an old non-SCI-related injury (old tibia-fibula fracture with peripheral nerve damage), with the motor score of that specific myotome impacting the determination of the AIS (Fig. 1)? In this case, does the AIS need to be recorded as “ND”?

#### Response

From a clinical perspective, the left L5 myotome can be considered as “normal for classification purposes”. This is documented on the worksheet by adding an asterisk (*) to the left L5 motor score and providing the reason for the asterisk (*) and how to handle it for classification as additional information in the comments box. As such, even though there is an abnormal motor score (1*) in left L5, this muscle is considered as a “normal” grade (= 5) for classification. Since this individual example has a NLI at C5, a motor score of ≥3/5 in ≥9 of the remaining myotomes below the NLI (18 of them) would lead to an AIS classification of AIS D, and less than 9 would lead to an AIS classification of AIS C. In this case scenario, there are nine key muscles below the NLI that have a strength of at least 3/5, including this L5 myotome. As such, the classification would be designated as an AIS D. Importantly, since this classification was made based upon a clinical assumption (the tagged score for the left L5 key muscle group), the designation of AIS D should be tagged (AIS D*). While for classification purposes the ‘*’-tagged motor score is replaced by a normal score this is not true for calculating the sum motor score of the left side. The sum scores are always based on the examined score. This results in a total motor score on the left side of 43.

A challenge in this case could occur if the examiner documented in the comment box that the 1* for the left L5 myotome—is “not considered as normal”. While this determines that the score is not considered a 5, the 1* could represent a score of any grade from 1 to 4. In this case, the grade of the myotome would make a difference in AIS classification. Specifically, if the 1* would be considered a grade of a 1 or 2, then the classification would be an AIS C (since less than 50% of the key muscles below the NLI would be considered to have a strength of ≥3/5), and if a 3 or 4, then an AIS D, as described above. Since either of these two scenarios are possible if marked as “not considered normal”, the appropriate designation for the AIS would be documented as “ND*” (not determinable), with the tagged grade signifying that this grade is based upon a tagged score in a key muscle.

### Challenging case 2

**Fig. 2:**
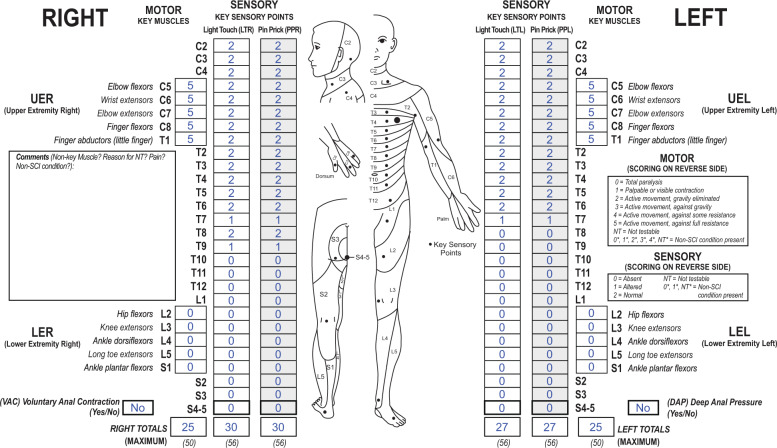
Case 2 with a complete SCI and preserved functions below the lesion level. This individual has a complete (AIS A) thoracic lesion with some sensory functions preserved below the NLI.

What is the right motor ZPP in a case where the sensory level is in the thoracic region, and sensation is intact at a level below the sensory level as seen in the example in the worksheet (Fig. 2)? As the rule is that the motor level defers to the sensory level in the thoracic area, would the right motor ZPP be T8 or T6?

#### Response

In the case presented (Fig. 2), the sensory level is T6, and the motor level (as well as the NLI) is also T6. The motor level defers to the sensory level since all key muscle functions at T1 and above are all intact. The sensory ZPP on the right is T9, however, the motor ZPP on the right remains at T6. Even though there is intact sensation at T8 on the right, one does not infer that motor function at T8 is also normal. In 2011, the rule for motor ZPP was clarified that “motor function does NOT follow sensory function in recording ZPP” [8]. This remained unchanged in the 2019 revision [4]. The “caudal extent of the motor ZPP must be based on the presence of *voluntary* muscle contraction below the motor level”. While the motor level defers to the sensory level in the regions where there is no key muscle function to test (C1–C4, T2–L1, S2-S4/5), motor ZPP does not defer to the sensory ZPP. In this case, the defined motor level is T6, with no apparent voluntary muscle action in key muscles below that T6 right motor level, therefore the right motor ZPP is T6.

### Challenging case 3

**Fig. 3:**
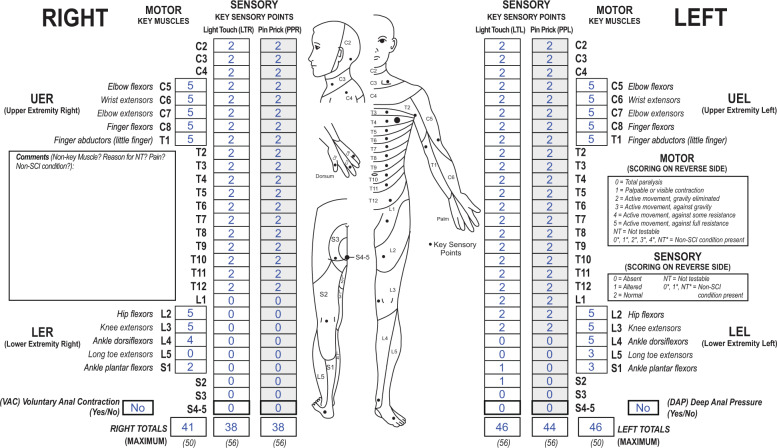
Case 3 of an ambulatory person with a complete SCI. This individual has substantial motor functions preserved below the motor level, but no sacral sparing of sensory or motor functions.

How would you classify a case with a neurological level of T12 where there is a significant amount of motor sparing more than three levels below the motor level on each side but the individual does not have sacral sparing of sensory and motor functions (see Fig. 3)? Is the sacral sparing definition still used and this individual classified as complete (AIS A) even if he or she is able to walk?

#### Response

Since there is no sacral sparing present in this case scenario, including light touch and pin sensation at S4-5, DAP, or voluntary anal contraction (VAC), this individual would be classified as a neurologically complete injury (AIS A). While it may seem counterintuitive that a person with a neurological complete injury can ambulate, the purpose of ISNCSCI is the determination of the neurological level and severity of a SCI and is not intended to be a functional measure. The constellation of “AIS D-like” capabilities in terms of walking with a classification of AIS A therefore can occur, although these cases are uncommon. One report found that this occurred in 3.2% of the overall AIS A population within the first year after injury [9]. For this specific case based upon the level of injury, one could consider that this may represent a cauda equina injury with some lower motor neuron damage findings especially of the anal sphincter, although this designation does not impact whether the injury is classified as neurologically complete or incomplete. Because of missing sensory and motor function in the lowest sacral segments, sensory (right T12, left S2) and motor ZPPs (right S1, left S1) are given and allow for characterization of the extent of preserved functions below the NLI of T12.

### Challenging case 4

**Fig. 4:**
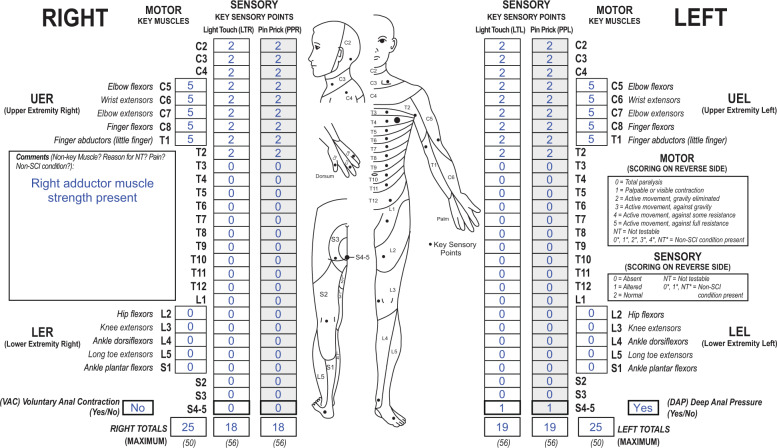
Case 4 with preserved sensory function in lowest sacral segments and present motor function in a non-key muscle below the lesion level. This individual has  a high-thoracic lesion with spared sensory function in S4-5 on the left side and motor function in a non-key muscle (hip adductor) on the right side.

How to classify an individual with a T2 sensory and motor level, with sparing of light touch and pin prick sensation on the left side only at the S4-5 dermatome and DAP, with no key muscle functions spared in bilateral lower extremities nor VAC, but the presence of voluntary right adductor muscle strength is noted (see Fig. 4)?

#### Response

The motor and sensory level as well as the NLI are T2. The presence of DAP, and left LT and PP sensation at S4-5, allows for classification as at least an AIS grade B. While there is no VAC present, the presence of the right adductor muscle, whose innervation (L2) is more than three segments below the motor level T2, allows this case scenario of a sensory incomplete SCI to meet the criteria for classification as an AIS grade C.

In this case, while sensory ZPPs on both sides are not applicable because of the presence of DAP, motor ZPPs are defined due to the missing VAC. Because preserved non-key muscle function led to an AIS C classification, L2 as the most distal spinal segment with preserved motor function is recorded as the motor ZPP (L2) on the right side. It should be emphasized that the most distal non-key muscle function is only recorded as the motor ZPP in those exceptional situations when the non-key muscle function(s) are used to determine an AIS C classification. On the left side, T2 is recorded as the motor ZPP.

### Challenging case 5

**Fig. 5:**
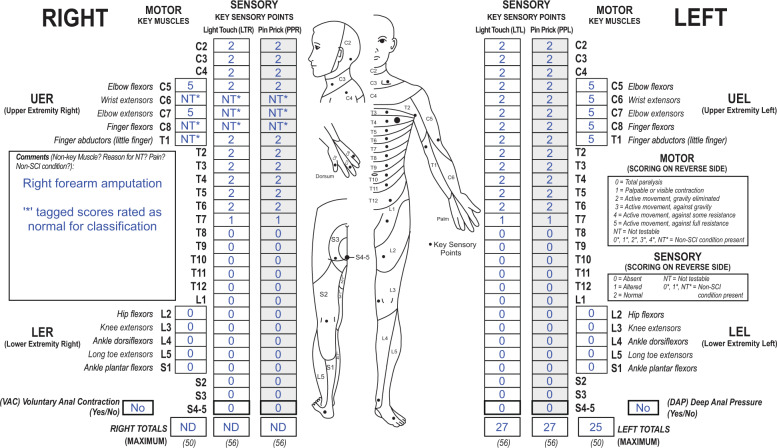
Case 5 with a non-SCI condition above the lesion level. This individual has a mid-thoracic lesion and a transradial amputation of the right upper extremity. Based on the clinical assumption, these asterisk (*)-tagged NT scores on the right arm should be considered as normal during classification, which is noted in the comments box.

In a case scenario where a patient’s right upper extremity underwent a transradial amputation below the level of the elbow, the key muscles and key sensory points distal to the elbow cannot be tested and would be graded as NT. How would you classify an individual with this amputation, who otherwise presents with what appears to be a T6 level (see Fig. 5)?

#### Response

The motor and sensory scores of those spinal segments affected of the right upper extremity secondary to the forearm amputation should be documented as NT*. The reason for the tagged scores is due to the impairment (the amputation) not being SCI related. In the comments box, the reason for the NTs and the tagged (‘*’) scores are documented, as well as whether the scores for the myotomes and dermatomes should be documented as normal for classification. Since the remaining aspects of the neurological picture reveal a T6 level of injury, the clinical decision is made that these myotomes and dermatomes would grade normal for classification. As such, the right motor and sensory level is graded as T6*, to follow the rule that levels changed because of tagged scores, should receive a tag. While the left motor and sensory levels would be documented as T6, and not require a tag, the NLI is documented as T6*, because the right motor and sensory levels of T6* are based on clinical assumptions and so does the single NLI.

The complete classification results of the five challenging cases can be found in the Supplementary Figs. 1–5.

## Discussion

These challenging cases offer reinforcement to a number of important concepts in classifying SCI. This includes some of the most recent revisions in the ISNCSCI in 2019 utilizing tagged scores, reinforcing complicated concepts with the ZPP, reinforcing the rules of the sacral sparing definition of a complete injury, how to use non-key muscles in classifying incomplete injuries, and how to classify an individual with an amputation above the SCI lesion.

Over the years, the ISNCSCI has undergone revisions and updates that are aimed to improve the classification based upon feedback from professionals in the SCI community. The reinforcement of some older recommendations, as well as the more recent introduction of the non-SCI taxonomy, we believe facilitates better documentation of challenging cases that are found clinically. Review of InSTep (https://asia-spinalinjury.org/instep/) and continued training is of utmost importance for consistent usage of these Standards. The ASIA International Standards Committee invites the SCI community to share more challenging cases to continuously improve the ISNCSCI and the documentation of the neurological findings.

## Conclusion

Presenting cases with challenging classifications, along with responses and explanations, will assist professionals serving the SCI population to better understand and utilize the ISNCSCI classification system. As the ISNCSCI evolves over time, such resources are important to clarify inquiries from the SCI community and to understand the rationale for revisions.

## Supplementary information

Supplementary Figures 1-5
