# Supplementary material for: Classification challenges of the 2019 revised International Standards for Neurological Classification of Spinal Cord Injury (ISNCSCI)
Source: Spinal Cord. 2021 Jun 4;60(1):11–7. doi: 10.1038/s41393-021-00648-y (PMC8737267; doi:10.1038/s41393-021-00648-y)
Supplement: Supplementary file 1 — Supplementary Figures 1-5 [file 41393_2021_648_MOESM1_ESM.pdf]

S. Kirshblum, M. Schmidt Read, and R. Rupp, Classification challenges of the 2019 revised International Standards for Neurological Classification of Spinal Cord Injury (ISNCSCI), *Spinal Cord*, 2021

This supplement contains the complete classification results of the five cases with classification challenges presented in the manuscript "Classification challenges of the 2019 revised International Standards for Neurological Classification of Spinal Cord Injury (ISNCSCI)" from S. Kirshblum, M. Schmidt Read and R. Rupp.

Supplementary figure 1: Complete classification of case 1 with unimpaired sensory function on the left side and a motor impairment of the left long toe extensor due to an old tibia fracture documented as 1* in the left L5 segment.

RIGHT / **LEFT**

RIGHT Motor Key Muscles		Motor	Sensory Light Touch (LTR)	Sensory Pin Prick (PPR)	Level	Sensory Light Touch (LTL)	Sensory Pin Prick (PPL)	Motor	LEFT Motor Key Muscles
	C2		2	2	C2	2	2		
	C3		2	2	C3	2	2		
	C4		2	2	C4	2	2		
UER (Upper Extremity Right): Elbow flexors	C5	5	2	2	C5	2	2	5	C5 Elbow flexors — UEL (Upper Extremity Left)
Wrist extensors	C6	2	1	1	C6	2	2	5	C6 Wrist extensors
Elbow extensors	C7	2	1	1	C7	2	2	5	C7 Elbow extensors
Finger flexors	C8	1	1	1	C8	2	2	4	C8 Finger flexors
Finger abductors (little finger)	T1	0	1	1	T1	2	2	3	T1 Finger abductors (little finger)
	T2		1	1	T2	2	2		
	T3		1	1	T3	2	2		
	T4		1	1	T4	2	2		
	T5		1	1	T5	2	2		
	T6		1	1	T6	2	2		
	T7		1	1	T7	2	2		
	T8		1	1	T8	2	2		
	T9		1	1	T9	2	2		
	T10		1	1	T10	2	2		
	T11		1	1	T11	2	2		
	T12		1	1	T12	2	2		
	L1		1	1	L1	2	2		
LER (Lower Extremity Right): Hip flexors	L2	1	1	1	L2	2	2	5	L2 Hip flexors — LEL (Lower Extremity Left)
Knee extensors	L3	2	1	1	L3	2	2	5	L3 Knee extensors
Ankle dorsiflexors	L4	0	1	1	L4	2	2	5	L4 Ankle dorsiflexors
Long toe extensors	L5	1	1	1	L5	2	2	1*	L5 Long toe extensors
Ankle plantar flexors	S1	2	1	1	S1	2	2	5	S1 Ankle plantar flexors
	S2		1	1	S2	2	2		
	S3		1	1	S3	2	2		
(VAC) Voluntary Anal Contraction (Yes/No): Yes	S4-5		1	1	S4-5	2	2		(DAP) Deep Anal Pressure (Yes/No): Yes
RIGHT TOTALS		16	32	32		56	56	43	LEFT TOTALS
(MAXIMUM)		(50)	(56)	(56)		(56)	(56)	(50)	(MAXIMUM)

Comments (Non-key Muscle? Reason for NT? Pain? Non-SCI condition?):
Old left tibia/fibula fracture

Left L5 motor score considered normal for classification purposes

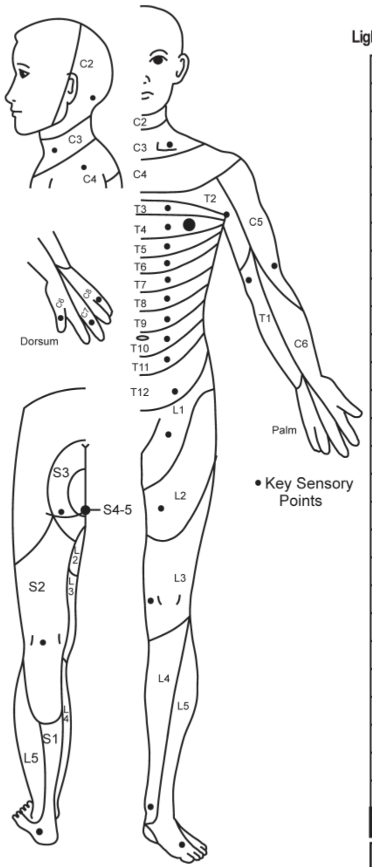

MOTOR (SCORING ON REVERSE SIDE)
0 = Total paralysis
1 = Palpable or visible contraction
2 = Active movement, gravity eliminated
3 = Active movement, against gravity
4 = Active movement, against some resistance
5 = Active movement, against full resistance
NT = Not testable
0*, 1*, 2*, 3*, 4*, NT* = Non-SCI condition present

SENSORY (SCORING ON REVERSE SIDE)
0 = Absent
1 = Altered
2 = Normal
NT = Not testable
0*, 1*, NT* = Non-SCI condition present

MOTOR SUBSCORES

UER	+UEL	= UEMS TOTAL	LER	+ LEL	= LEMS TOTAL
10	22	32	6	21	27
MAX (25)	(25)	(50)	MAX (25)	(25)	(50)

SENSORY SUBSCORES

LTR	+ LTL	= LT TOTAL	PPR	+ PPL	= PP TOTAL
32	56	88	32	56	88
MAX (56)	(56)	(112)	MAX (56)	(56)	(112)

NEUROLOGICAL LEVELS (Steps 1- 6 for classification as on reverse)

	R	L
1. SENSORY	C5	INT
2. MOTOR	C5	C8

3. NEUROLOGICAL LEVEL OF INJURY (NLI): C5

4. COMPLETE OR INCOMPLETE? (Incomplete = Any sensory or motor function in S4-5): Inc

5. ASIA IMPAIRMENT SCALE (AIS): D*

6. ZONE OF PARTIAL PRESERVATION (In injuries with absent motor OR sensory function in S4-5 only) — Most caudal levels with any innervation

	R	L
SENSORY	NA	NA
MOTOR	NA	NA

S. Kirshblum, M. Schmidt Read, and R. Rupp, Classification challenges of the 2019 revised International Standards for Neurological Classification of Spinal Cord Injury (ISNCSCI), *Spinal Cord*, 2021

Supplementary figure 2: Complete classification of case 2 of a complete (AIS A) thoracic lesion with some sensory functions preserved below the NLI.

RIGHT

Key muscles (Right)	Level	MOTOR	SENSORY Light Touch (LTR)	SENSORY Pin Prick (PPR)
	C2		2	2
	C3		2	2
	C4		2	2
Elbow flexors	C5	5	2	2
Wrist extensors	C6	5	2	2
Elbow extensors	C7	5	2	2
Finger flexors	C8	5	2	2
Finger abductors (little finger)	T1	5	2	2
	T2		2	2
	T3		2	2
	T4		2	2
	T5		2	2
	T6		2	2
	T7		1	1
	T8		2	2
	T9		1	1
	T10		0	0
	T11		0	0
	T12		0	0
	L1		0	0
Hip flexors	L2	0	0	0
Knee extensors	L3	0	0	0
Ankle dorsiflexors	L4	0	0	0
Long toe extensors	L5	0	0	0
Ankle plantar flexors	S1	0	0	0
	S2		0	0
	S3		0	0
	S4-5		0	0
RIGHT TOTALS (MAXIMUM)		25 (50)	30 (56)	30 (56)

UER (Upper Extremity Right)

LER (Lower Extremity Right)

Comments (Non-key Muscle? Reason for NT? Pain? Non-SCI condition?):

(VAC) Voluntary Anal Contraction (Yes/No): No

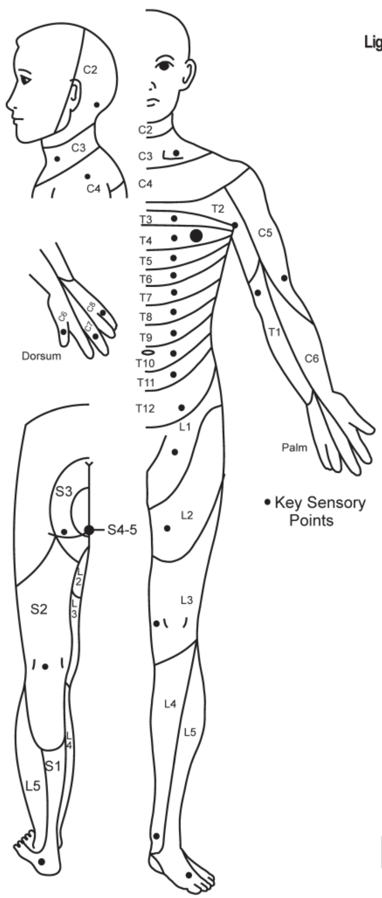

LEFT

Level	SENSORY Light Touch (LTL)	SENSORY Pin Prick (PPL)	MOTOR	Key muscles (Left)
C2	2	2		
C3	2	2		
C4	2	2		
C5	2	2	5	Elbow flexors
C6	2	2	5	Wrist extensors
C7	2	2	5	Elbow extensors
C8	2	2	5	Finger flexors
T1	2	2	5	Finger abductors (little finger)
T2	2	2		
T3	2	2		
T4	2	2		
T5	2	2		
T6	2	2		
T7	1	1		
T8	0	0		
T9	0	0		
T10	0	0		
T11	0	0		
T12	0	0		
L1	0	0		
L2	0	0	0	Hip flexors
L3	0	0	0	Knee extensors
L4	0	0	0	Ankle dorsiflexors
L5	0	0	0	Long toe extensors
S1	0	0	0	Ankle plantar flexors
S2	0	0		
S3	0	0		
S4-5	0	0		
LEFT TOTALS (MAXIMUM)	27 (56)	27 (56)	25 (50)	

UEL (Upper Extremity Left)

LEL (Lower Extremity Left)

(DAP) Deep Anal Pressure (Yes/No): No

MOTOR (SCORING ON REVERSE SIDE)

0 = Total paralysis
1 = Palpable or visible contraction
2 = Active movement, gravity eliminated
3 = Active movement, against gravity
4 = Active movement, against some resistance
5 = Active movement, against full resistance
NT = Not testable
0, 1*, 2*, 3*, 4*, NT* = Non-SCI condition present*

SENSORY (SCORING ON REVERSE SIDE)

0 = Absent
1 = Altered
2 = Normal
NT = Not testable
0, 1*, NT* = Non-SCI condition present*

MOTOR SUBSCORES

UER	+UEL	= UEMS TOTAL	LER	+ LEL	= LEMS TOTAL
25	25	50	0	0	0
MAX (25)	(25)	(50)	MAX (25)	(25)	(50)

SENSORY SUBSCORES

LTR	+ LTL	= LT TOTAL	PPR	+ PPL	= PP TOTAL
30	27	57	30	27	57
MAX (56)	(56)	(112)	MAX (56)	(56)	(112)

NEUROLOGICAL LEVELS
Steps 1- 6 for classification as on reverse

	R	L
1. SENSORY	T6	T6
2. MOTOR	T6	T6

3. NEUROLOGICAL LEVEL OF INJURY (NLI): T6

4. COMPLETE OR INCOMPLETE?: Com
Incomplete = Any sensory or motor function in S4-5

5. ASIA IMPAIRMENT SCALE (AIS): A

6. ZONE OF PARTIAL PRESERVATION
(In injuries with absent motor OR sensory function in S4-5 only)
Most caudal levels with any innervation

	R	L
SENSORY	T9	T7
MOTOR	T6	T6

S. Kirshblum, M. Schmidt Read, and R. Rupp, Classification challenges of the 2019 revised International Standards for Neurological Classification of Spinal Cord Injury (ISNCSCI), *Spinal Cord*, 2021

Supplementary figure 3: Complete classification of case 3 of an ambulatory person with a substantial preservation of motor functions below the motor level, but no sacral sparing of sensory and motor functions.

RIGHT Key muscles	Level	Right Motor	Light Touch (LTR)	Pin Prick (PPR)	Light Touch (LTL)	Pin Prick (PPL)	Left Motor	Level	LEFT Key muscles
	C2		2	2	2	2		C2	
	C3		2	2	2	2		C3	
	C4		2	2	2	2		C4	
Elbow flexors	C5	5	2	2	2	2	5	C5	Elbow flexors
Wrist extensors	C6	5	2	2	2	2	5	C6	Wrist extensors
Elbow extensors	C7	5	2	2	2	2	5	C7	Elbow extensors
Finger flexors	C8	5	2	2	2	2	5	C8	Finger flexors
Finger abductors (little finger)	T1	5	2	2	2	2	5	T1	Finger abductors (little finger)
	T2		2	2	2	2		T2	
	T3		2	2	2	2		T3	
	T4		2	2	2	2		T4	
	T5		2	2	2	2		T5	
	T6		2	2	2	2		T6	
	T7		2	2	2	2		T7	
	T8		2	2	2	2		T8	
	T9		2	2	2	2		T9	
	T10		2	2	2	2		T10	
	T11		2	2	2	2		T11	
	T12		2	2	2	2		T12	
	L1		0	0	2	2		L1	
Hip flexors	L2	5	0	0	2	2	5	L2	Hip flexors
Knee extensors	L3	5	0	0	2	2	5	L3	Knee extensors
Ankle dorsiflexors	L4	4	0	0	0	0	5	L4	Ankle dorsiflexors
Long toe extensors	L5	0	0	0	0	0	3	L5	Long toe extensors
Ankle plantar flexors	S1	2	0	0	1	0	3	S1	Ankle plantar flexors
	S2		0	0	1	0		S2	
	S3		0	0	0	0		S3	
	S4-5		0	0	0	0		S4-5	
RIGHT TOTALS		41	38	38	46	44	46		LEFT TOTALS
(MAXIMUM)		(50)	(56)	(56)	(56)	(56)	(50)		(MAXIMUM)

UER (Upper Extremity Right); UEL (Upper Extremity Left); LER (Lower Extremity Right); LEL (Lower Extremity Left)

Comments (Non-key Muscle? Reason for NT? Pain? Non-SCI condition?):

(VAC) Voluntary Anal Contraction (Yes/No): No

(DAP) Deep Anal Pressure (Yes/No): No

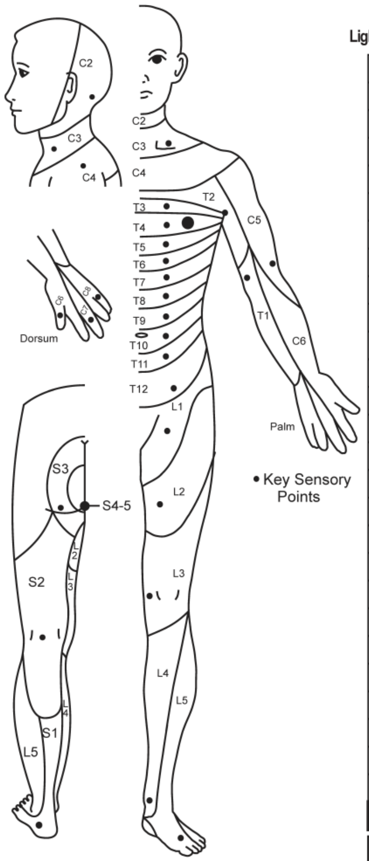

MOTOR (SCORING ON REVERSE SIDE)

0 = Total paralysis
1 = Palpable or visible contraction
2 = Active movement, gravity eliminated
3 = Active movement, against gravity
4 = Active movement, against some resistance
5 = Active movement, against full resistance
NT = Not testable
0*, 1*, 2*, 3*, 4*, NT* = Non-SCI condition present

SENSORY (SCORING ON REVERSE SIDE)

0 = Absent
1 = Altered
2 = Normal
NT = Not testable
0*, 1*, NT* = Non-SCI condition present

MOTOR SUBSCORES

UER 25 + UEL 25 = UEMS TOTAL 50 (MAX (25), (25), (50))

LER 16 + LEL 21 = LEMS TOTAL 37 (MAX (25), (25), (50))

SENSORY SUBSCORES

LTR 38 + LTL 46 = LT TOTAL 84 (MAX (56), (56), (112))

PPR 38 + PPL 44 = PP TOTAL 82 (MAX (56), (56), (112))

NEUROLOGICAL LEVELS (Steps 1-6 for classification as on reverse)	R	L
1. SENSORY	T12	L3
2. MOTOR	T12	L5

3. NEUROLOGICAL LEVEL OF INJURY (NLI): T12

4. COMPLETE OR INCOMPLETE? (Incomplete = Any sensory or motor function in S4-5): Com

5. ASIA IMPAIRMENT SCALE (AIS): A

6. ZONE OF PARTIAL PRESERVATION (Most caudal levels with any innervation) (In injuries with absent motor OR sensory function in S4-5 only)	R	L
SENSORY	T12	S2
MOTOR	S1	S1

Supplementary figure 4: Complete Classification of case 4 of a high thoracic lesion with spared sensory function in S4-5 on the left side and motor function in a non-key muscle (hip adductor) on the right side.

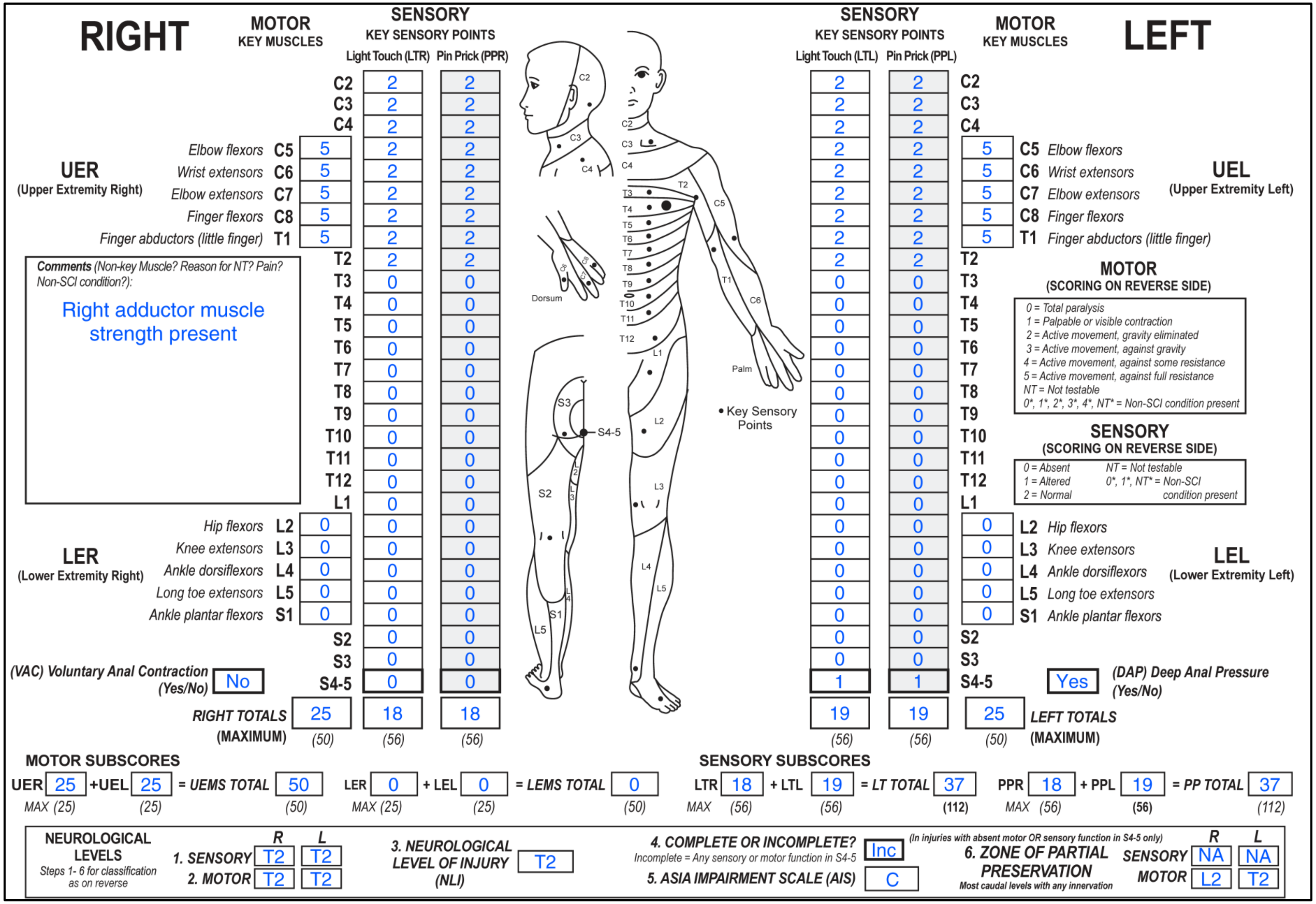

RIGHT | **LEFT**

RIGHT Motor Key Muscles	Level	Right Motor	Sensory Light Touch (LTR)	Sensory Pin Prick (PPR)	Sensory Light Touch (LTL)	Sensory Pin Prick (PPL)	Level	Left Motor	LEFT Motor Key Muscles
	C2		2	2	2	2	C2		
	C3		2	2	2	2	C3		
	C4		2	2	2	2	C4		
UER (Upper Extremity Right): Elbow flexors	C5	5	2	2	2	2	C5	5	Elbow flexors — UEL (Upper Extremity Left)
Wrist extensors	C6	5	2	2	2	2	C6	5	Wrist extensors
Elbow extensors	C7	5	2	2	2	2	C7	5	Elbow extensors
Finger flexors	C8	5	2	2	2	2	C8	5	Finger flexors
Finger abductors (little finger)	T1	5	2	2	2	2	T1	5	Finger abductors (little finger)
	T2		2	2	2	2	T2		
	T3		0	0	0	0	T3		
	T4		0	0	0	0	T4		
	T5		0	0	0	0	T5		
	T6		0	0	0	0	T6		
	T7		0	0	0	0	T7		
	T8		0	0	0	0	T8		
	T9		0	0	0	0	T9		
	T10		0	0	0	0	T10		
	T11		0	0	0	0	T11		
	T12		0	0	0	0	T12		
	L1		0	0	0	0	L1		
LER (Lower Extremity Right): Hip flexors	L2	0	0	0	0	0	L2	0	Hip flexors — LEL (Lower Extremity Left)
Knee extensors	L3	0	0	0	0	0	L3	0	Knee extensors
Ankle dorsiflexors	L4	0	0	0	0	0	L4	0	Ankle dorsiflexors
Long toe extensors	L5	0	0	0	0	0	L5	0	Long toe extensors
Ankle plantar flexors	S1	0	0	0	0	0	S1	0	Ankle plantar flexors
	S2		0	0	0	0	S2		
	S3		0	0	0	0	S3		
(VAC) Voluntary Anal Contraction (Yes/No): No	S4-5		0	0	1	1	S4-5		(DAP) Deep Anal Pressure (Yes/No): Yes
RIGHT TOTALS		25	18	18	19	19		25	LEFT TOTALS
(MAXIMUM)		(50)	(56)	(56)	(56)	(56)		(50)	(MAXIMUM)

Comments (Non-key Muscle? Reason for NT? Pain? Non-SCI condition?): Right adductor muscle strength present

MOTOR (SCORING ON REVERSE SIDE)

0 = Total paralysis
1 = Palpable or visible contraction
2 = Active movement, gravity eliminated
3 = Active movement, against gravity
4 = Active movement, against some resistance
5 = Active movement, against full resistance
NT = Not testable
0*, 1*, 2*, 3*, 4*, NT* = Non-SCI condition present

SENSORY (SCORING ON REVERSE SIDE)

0 = Absent
1 = Altered
2 = Normal
NT = Not testable
0*, 1*, NT* = Non-SCI condition present

MOTOR SUBSCORES

UER 25 + UEL 25 = UEMS TOTAL 50 — MAX (25) (25) (50)

LER 0 + LEL 0 = LEMS TOTAL 0 — MAX (25) (25) (50)

SENSORY SUBSCORES

LTR 18 + LTL 19 = LT TOTAL 37 — MAX (56) (56) (112)

PPR 18 + PPL 19 = PP TOTAL 37 — MAX (56) (56) (112)

NEUROLOGICAL LEVELS (Steps 1-6 for classification as on reverse)

	R	L
1. SENSORY	T2	T2
2. MOTOR	T2	T2

3. NEUROLOGICAL LEVEL OF INJURY (NLI): T2

4. COMPLETE OR INCOMPLETE? Inc (Incomplete = Any sensory or motor function in S4-5)

5. ASIA IMPAIRMENT SCALE (AIS): C

6. ZONE OF PARTIAL PRESERVATION (In injuries with absent motor OR sensory function in S4-5 only) — Most caudal levels with any innervation

	R	L
SENSORY	NA	NA
MOTOR	L2	T2

S. Kirshblum, M. Schmidt Read, and R. Rupp, Classification challenges of the 2019 revised International Standards for Neurological Classification of Spinal Cord Injury (ISNCSCI), *Spinal Cord*, 2021

Supplementary figure 5: Complete classification of case 5 with a mid-thoracic lesion and a transradial amputation of the right upper extremity.

RIGHT

Key muscles	Level	MOTOR	SENSORY Light Touch (LTR)	SENSORY Pin Prick (PPR)
	C2		2	2
	C3		2	2
	C4		2	2
Elbow flexors	C5	5	2	2
Wrist extensors	C6	NT*	NT*	NT*
Elbow extensors	C7	5	NT*	NT*
Finger flexors	C8	NT*	NT*	NT*
Finger abductors (little finger)	T1	NT*	2	2
	T2		2	2
	T3		2	2
	T4		2	2
	T5		2	2
	T6		2	2
	T7		1	1
	T8		0	0
	T9		0	0
	T10		0	0
	T11		0	0
	T12		0	0
	L1		0	0
Hip flexors	L2	0	0	0
Knee extensors	L3	0	0	0
Ankle dorsiflexors	L4	0	0	0
Long toe extensors	L5	0	0	0
Ankle plantar flexors	S1	0	0	0
	S2		0	0
	S3		0	0
	S4-5		0	0
RIGHT TOTALS (MAXIMUM)		ND (50)	ND (56)	ND (56)

UER (Upper Extremity Right)

LER (Lower Extremity Right)

(VAC) Voluntary Anal Contraction (Yes/No): No

Comments (Non-key Muscle? Reason for NT? Pain? Non-SCI condition?):
Right forearm amputation
'*' tagged scores rated as normal for classification

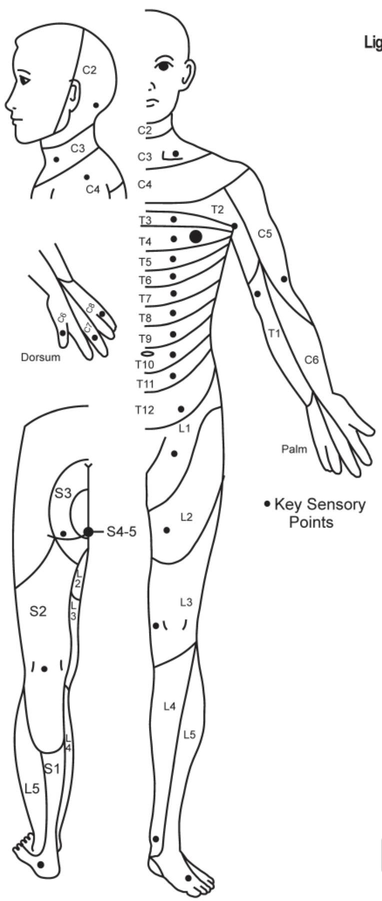

LEFT

Level	SENSORY Light Touch (LTL)	SENSORY Pin Prick (PPL)	MOTOR	Key muscles
C2	2	2		
C3	2	2		
C4	2	2		
C5	2	2	5	Elbow flexors
C6	2	2	5	Wrist extensors
C7	2	2	5	Elbow extensors
C8	2	2	5	Finger flexors
T1	2	2	5	Finger abductors (little finger)
T2	2	2		
T3	2	2		
T4	2	2		
T5	2	2		
T6	2	2		
T7	1	1		
T8	0	0		
T9	0	0		
T10	0	0		
T11	0	0		
T12	0	0		
L1	0	0		
L2	0	0	0	Hip flexors
L3	0	0	0	Knee extensors
L4	0	0	0	Ankle dorsiflexors
L5	0	0	0	Long toe extensors
S1	0	0	0	Ankle plantar flexors
S2	0	0		
S3	0	0		
S4-5	0	0		
LEFT TOTALS (MAXIMUM)	27 (56)	27 (56)	25 (50)	

UEL (Upper Extremity Left)

LEL (Lower Extremity Left)

(DAP) Deep Anal Pressure (Yes/No): No

MOTOR (SCORING ON REVERSE SIDE)

0 = Total paralysis
1 = Palpable or visible contraction
2 = Active movement, gravity eliminated
3 = Active movement, against gravity
4 = Active movement, against some resistance
5 = Active movement, against full resistance
NT = Not testable
0, 1*, 2*, 3*, 4*, NT* = Non-SCI condition present*

SENSORY (SCORING ON REVERSE SIDE)

0 = Absent
1 = Altered
2 = Normal
NT = Not testable
0, 1*, NT* = Non-SCI condition present*

MOTOR SUBSCORES

UER	+ UEL	= UEMS TOTAL	LER	+ LEL	= LEMS TOTAL
ND	25	ND	0	0	0
MAX (25)	(25)	(50)	MAX (25)	(25)	(50)

SENSORY SUBSCORES

LTR	+ LTL	= LT TOTAL	PPR	+ PPL	= PP TOTAL
ND	27	ND	ND	27	ND
MAX (56)	(56)	(112)	MAX (56)	(56)	(112)

NEUROLOGICAL LEVELS (Steps 1- 6 for classification as on reverse)

	R	L
1. SENSORY	T6*	T6
2. MOTOR	T6*	T6

3. NEUROLOGICAL LEVEL OF INJURY (NLI): T6*

4. COMPLETE OR INCOMPLETE? (Incomplete = Any sensory or motor function in S4-5): Com

5. ASIA IMPAIRMENT SCALE (AIS): A

6. ZONE OF PARTIAL PRESERVATION (In injuries with absent motor OR sensory function in S4-5 only) — Most caudal levels with any innervation

	R	L
SENSORY	T7	T7
MOTOR	T6*	T6
